# Decentralisation and Management of Human Resource for Health in the Health System of Ghana: A Decision Space Analysis

**DOI:** 10.15171/ijhpm.2018.88

**Published:** 2018-09-23

**Authors:** Anthony Mwinkaara Sumah, Leonard Baatiema

**Affiliations:** ^1^Ghana Health Service, Upper West Regional Health Directorate, Wa, Ghana.; ^2^Regional Institute for Population Studies, University of Ghana, Legon-Accra, Ghana.; ^3^School of Allied and Public Health, Faculty of Health Sciences, Australian Catholic University, Sydney, NSW, Australia.

**Keywords:** Decentralisation, Decision Space, Human Resource Management, Health System, Ghana

## Abstract

**Background:** The implications of decentralisation on human resource for health management has not received adequate research attention despite the presupposition that the concept of decentralisation leads to the transfer of management authority and discretion for human resource management from national levels to subnational levels. This study aims at investigating the extent to which decentralisation practice transfers management autonomy and discretion to subnational units, and the effect of the level of decision space on human resource management in the health sector.

**Methods:** A mixed methods study design was adopted employing a cross-sectional survey and a document analysis. The respondents included health managers from the regional, district and hospital administrations as well as facility managers from the community-based health planning and services zones. A decision space framework was employed to measure management autonomy and discretion at various management levels of the study region. For the quantitative data, descriptive statistical analysis was used to analyse and report the data whilst the qualitative data was contentanalysed.

**Results:** The study reported that in practice, management authority for core human resource functions such as recruitment, remuneration, personnel training and development are centralised rather than transferred to the subnational units. It further reveals that authority diminishes along the management continuum from the national to the community level. Decentralisation was however found to have led to greater autonomy in technical supervision and performance appraisal. The study also reported the existence of discrepancy between the wide decision space for performance assessment through technical supervision and performance appraisal exercised by managers at the subnational level and a rather limited discretion for providing incentives or rewards to staff.

**Conclusion:** The practice of decentralisation in the Ghanaian health sector is more apparent than real. The limited autonomy and discretion in the management of human resource at the subnational units have potential adverse implications on effective recruitment, retention, development and distribution of health personnel. Therefore, further decision space is required at the subnational level to enhance effective and efficient management of human resource to attain the health sector objectives.

## Background

Arguably, human resource is one of the most important resource in a labour-intensive health sector. Consequently, health sector organisational performance is inextricably linked to the effectiveness of its workforce which is further a function of the existing human resource management policies and practices.^1,2^ In an effort to improve health systems performance, major health reforms have taken place globally and decentralisation is considered one of such reforms aimed at improving the efficiency and equity of health systems as well as responding to local preferences of populations.^3^ Besides the view that decentralisation is a means of responding to system-wide performance challenges, it is also argued that decentralisation is a means of improving performance in specific functional management areas such as human resources management.^1^

A considerable amount of literature has been published on the impact of decentralisation reforms on health systems. Much of this literature focussed on health system change and performance with emphasis on efficiency and equity in health service delivery and financing.^4-9^ As such, from the review of the relevant literature, the impact of decentralisation reforms on the management of human resource for health has not received adequate attention. Moreover, studies which have attempted to address this dearth of knowledge tended to focus on certain aspects such as human resource attraction and retention, policy restructuring, staff workload, human resource stock, quality of care, staff knowledge on decentralisation reforms and capacity of local authorities to implement decentralisation among others.^2,10-15^ Nonetheless, the findings from these studies regarding the effect of decentralisation are mixed. More importantly, although it is generally anticipated that decentralisation leads to the expansion of decision space at the local level,^10,16,17^ literature on the level of decision space available to local authorities in practice particularly with regards to human resource management remains poorly understood. The above situation persists notwithstanding the fact that, the control of human resource management in the health sector is a major factor in local decision space that has far reaching effects on health sector performance.^5^

Ghana is an example of a country that has institutionalised decentralisation as part its health sector reforms. Consequently, fiscal, administrative and political authority for the delivery of healthcare is expected to be transferred from the central Ministry of Health (MoH) to Ghana health service (GHS) and other alternate institutions in an effort to ensure efficiency, equity, service innovations, quality, transparency, and accountability in the health sector.^5,18^ Through the passing into law of GHS and Teaching Hospital Act 1996, (Act 525), Ghana established the legal framework to operationalise decentralisation in the health sector. The Act sought to delegate governance of healthcare from the central MoH to the GHS and teaching hospitals while applying deconcentration within the GHS and its subnational bodies. Two decades after its implementation, few studies^5,19^ have attempted to examine its implication on the performance of the health sector. To our knowledge, studies which provide a systematic understanding of how decentralisation has impacted on the management of human resource in Ghana’s health sector are limited. This study, therefore, seeks to investigate the amount of choice that is transferred from the central MoH to the subnational health systems and its attendant implications for the management of human resource. The study findings have the potential to provide useful insights on the effect of decentralisation on the management of human for health. Also, besides bridging this knowledge gap, the study will equally provide useful lessons for future decentralisation attempts or reforms in the health system in Ghana and beyond. To provide adequate background to examine the human resource management implications of decentralisation, the concepts of decentralisation and decision space are examined in detail in the ensuing paragraphs.

### Concept of Decentralisation

Decentralisation is conceptualised as the transfer of authorities from central government bodies to lower levels within the public sector or to autonomous institutions.^20^ Essentially 3 typologies of the concept exist in literature. As such decentralisation may be political, fiscal or administrative.^21^ Some researchers however propose a fourth typology – market decentralisation to account for the direction of public enterprise decentralisation in the form of privatisation and deregulation.^22^ Political decentralisation extends decision-making power governing public institutions to citizens at the local level while fiscal decentralisation relates to subnational ability to control financial resources, including revenue generation and allocation of funds.^21^ Administrative decentralisation on the other hand is the transfer of responsibility for the planning, financing and management of certain public functions from the central government and its agencies to field units of government agencies, subordinate units or levels of government, semi-autonomous public authorities or corporations, or area-wide, regional or functional authorities. From the perspective that the transfer of authority under administrative decentralisation may take several forms along a continuum of lesser and greater degrees, administrative decentralisation can be categorised into 3 main typologies or forms namely deconcentration, devolution and delegation. That is, administrative decentralisation may therefore be characterised as deconcentration of authorities from the central to local levels within a ministry or department of health, delegation of authorities to semi-autonomous bodies or devolution of responsibilities to autonomous or separate local governments.^21^ A fourth variation of decentralisation that has emerged in the literature is privatisation which involves the transfer of government functions to voluntary organisations or private enterprise.^20,23^

Depending on the existing political, public administration and health system structures in place, decentralisation may also take several forms across and within health systems or countries.^23^ They may vary based on size of the sub-national governments, level of decision-making autonomy or resource allocation responsibility. These variations have given reason to measure the practice of decentralisation, an underlying rationale for this study.

### Decentralisation in the Ghanaian Context

Decentralisation efforts in Ghana predates the attainment of independence from British rule.^24^ Concrete steps to institutionalise decentralisation as a preferred governance strategy however commence post-independence with the Local Government Act, Act 54 (1961). This piece of legislation built on previous ordinances to establish towns and municipalities, maintained distinction between local government and central government structures and operated dual hierarchical structures in parallel with central government structures.^24^ The current practice of decentralisation in Ghana however draws its legal framework from the Local Governance Act, 2016 (ACT 936). This act adopted devolution as the preferred Government of Ghana’s policy for governance. As such, the act provides for the creation of district assemblies and the devolution of power to the district assemblies as the highest political decision-making bodies in the districts. To this extent, Ghana has been organised into 10 regions with 216 districts. Local governance structure in the Ghanaian context comprises the district assemblies who are monitored and coordinated by the regional co-ordinating councils. Below the district assemblies are the area councils and the unit committees which form the last 2 levels of the governance structure in accordance with Act 936 (Republic of Ghana 2016).

Notwithstanding the adoption of the devolution as the preferred government of Ghana governance policy, decentralisation in Ghana’s health sector is at variance with devolution. The health sector rather saw a delegation from the MoH to the GHS and Teaching hospitals and a deconcentration within the GHS as provided by ACT 525. This process has led to the establishment of the GHS at the national, regional, district and sub-district levels giving rise to a 5-tier (national, regional, district, sub-district, and community-based health planning and services [CHPS] zones) governance structure currently in practice in the GHS. The variance in the forms of decentralisation practiced between the local government and the health sector has resulted in a mixed model of both devolution and deconcentration leading to unclear and in some instance contradictory reform efforts.^24^ The situation has also led to dual reporting lines for the GHS as it has to fulfil reporting obligations both to the hierarchy of the GHS and the district assembly system.^24^

### Conceptual Framework-Decision Space

The study relied on decision space framework developed by Bossert for analysing health sector decentralisation.^16^ Based on the central principle that decentralisation transfers authority, responsibility and resources for the management of healthcare from central MoH to regional and municipal governments as well as other autonomous institutions, this framework provides the means to analyse and compare in a consistent manner the amount of choice that is transferred from central institutions to institutions at the periphery of health systems. It therefore provides a mechanism for analysing decentralisation based on the set of functions and degrees of choice that are formally transferred to local officials. To measure the range of choice, decision space analysis is employed. In this framework, ‘decision space’ refers to the range of effective choice that is allowed by the central authorities to be utilised by the local authorities or alternatively, the ‘range of choice’ local decision-makers have available in a decentralised context.^5,16,25^ Beyond the choice that is allowed for decision-making at the local levels, the decision space framework further offers the opportunity to evaluate the kind of choices local officials make with their increased discretion and the implications these choices have on the performance of the health system through a decision space map.^16^ To estimate the degree of discretion or range of choices local authorities have over the various functions, the decision space frameworks illustrates this as either ‘narrow,’ ‘moderate,’ or and ‘wide’ (see decision map in Table 1).

**Table 1:** Map of Decision Space

**Functions**	**Range of Choice**		
	** Narrow**	** Moderate**	** Wide**
Finance			
Sources of revenue	→	→	→
Allocation of expenditures	→	→	→
Income from fees contracts	→	→	→
Service organization			
Hospital autonomy	→	→	→
Insurance plans	→	→	→
Payment mechanisms	→	→	→
Contracts with private providers	→	→	→
Required programs/norms	→	→	→
Human resources			
Salaries	→	→	→
Contracts	→	→	→
Civil service	→	→	→
Access rules			
Targeting	→	→	→
Government rules			
Facility boards	→	→	→
Health offices	→	→	→
Community participation	→	→	→

Source: Bossert 1998.

This paper adopts this approach because it provides the opportunity to disaggregate the various human resources functions over which local officials have a defined range of discretion and to examine the level of choice regarding these functions and how it impacts on the overall management of human resource. In this study we disaggregated the human resource function according to 3 categories (employment management, personnel administration and staff development) as espoused by a group of researchers^26^ and apply the decision map to these categories.

## Methods

### Study Design

The study is part of a larger mixed-methods design conducted between March and December 2013 to investigate the degree of decentralisation in GHS and its attendant impact on health sector performance. The use of qualitative and quantitative methods of inquiry are increasingly used in recent years due to their epistemological value and practical demands.^27-29^ To this, we collected data simultaneously and conducted the analysis as a strategy to triangulate and corroborate the findings from the 3 datasets.

This larger study design principally employed a cross sectional survey and document analysis. The cross-sectional survey was in 2 parts. The first utilised structured questionnaires to collect data from the relevant study participants on the discretion or authority available to them regarding human resource and other key management functions. The second part involved an in-depth interview of a key informant to elicit information on healthcare delivery relevant to human resources for health and other management functions covered by the study. This was done to ensure in-depth and rich data was collected from the perspective of a key decision-maker on the subject. The survey study design was favoured as most appropriate for this study due to its added strength of facilitating a broader understanding of human resources practices within a health sector.^30^ As a supplement to the empirical data, document analysis approach using a document content analysis method was conducted as the second part of this larger study to review all relevant national, regional and local health policy documents on this subject matter. This serves to validate and add on to the empirical data from the cross-sectional survey.

In this paper, drawing on insights from the cross-sectional survey, and document analysis of this larger study, we report the extent to which decentralisation practice transfers management autonomy and discretion to subnational units, and the effect of the level of decision space on human resource management in the health sector.

The study got approval from the Regional Director of Health Services through the Research Division of the Upper West Regional Health Service. The study was conducted according to sound ethical principles and guidelines as outlined in the Helsinki Declaration for Medical Research.^31^ Participants were informed about the study purpose and the voluntary nature of their participation prior to data collection. Anonymity of participants’ identity and data confidentiality were emphasised prior to all data collection sessions with the participants.

### Study Setting

The study was conducted in the Upper West Region, 1 of the 10 administrative regions in Ghana. The administrative and governance structure of health service in this region is also reflective of the other 9 regions and may share similar human resources for health characteristics. The governance structure for public health service in the study region is 4-tier made up of; a regional health administration at its apex, 11 district health administrations, 65 sub-districts and 166 CHPS zones. In addition, the region is made up of 6 service organisations comprising a regional hospital and 5 district hospitals. The region also contains private healthcare providers as well as health training institutions. These are not included in this study because of their multiple reporting structures which inhibit their direct classification under the purview of the regional health service. Figure provides details of the organisational structure.

**Figure:**
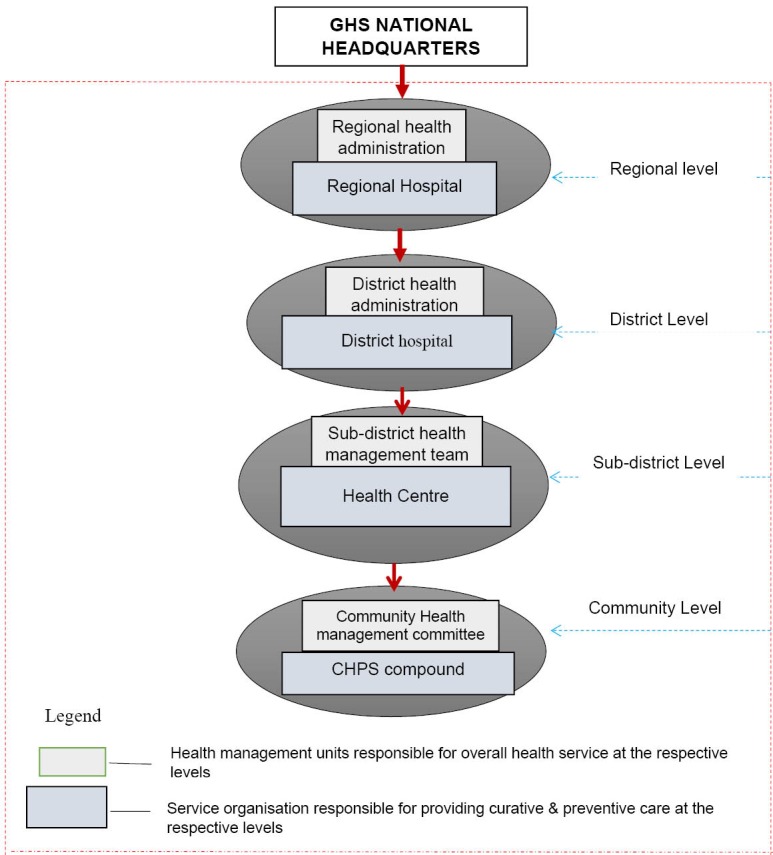
Organisational Structure of the Regional Health Service. Abbreviations: CHPS, community-based health planning and services; GHS, Ghana health service.
*Source:* Adapted from GHS organisational structure.

### Study Participants and Sampling Method

For the quantitative aspect of the survey, participants targeted included managers at the lower levels made up of, CHPS zones, sub-districts, district hospitals and health directorates. Participants were part of management involved in the daily operations and service delivery at the facility level, thus take key and strategic decisions related to the operations of the health facility. The second part of the survey involved conducting an in-depth interview was conducted on 1 participant who is the head of the regional health service with over 15 years of management experiences in the Ghanaian healthcare sector, thus most suitable to provide in-depth and insightful information.

A multi-staged sampling procedure was adopted for the survey. First, for participants in the cross-sectional study, the study used a purposive sampling technique to enlist them. Only heads of health facilities were enlisted in the study because of our exclusive focus on local actors responsible for the implementation of decentralised authority and responsibility. Here, all heads of regional and district health services as well as hospitals in the study area or persons acting in these positions were recruited. Given their small number, it was practical to enrol the regional health administration, all district health administrations and hospitals in the region. Second, regarding the sub districts, given the large sample size, we sampled the population using a standardised formula to estimate a representative sample. Stratified sampling was thereafter used to enlist the heads of the sub-district units and CHPS zones. Each district was considered as a stratum and the number of sub-district managers and CHPS zones were statistically determined to reflect the proportion of the sub-districts and CHPS zones in the district. Consequently, a simple random sampling was used to recruit participants. A purposive sampling technique was also employed to recruit 1 participant for the semi-structured interview.

### Study Instruments, Data Collection, and Analysis

A structured self-administered questionnaire and interview guide were developed based on the decision space framework and used to elicit responses from all study participants. The questionnaire had several sections seeking to understand decision space available to managers. The sections included level and practice of decentralisation regarding human resources, planning and budgeting, finance, governance and community participation, limitations to effective decentralisation and facilitators of effective decentralisation. The semi-structured interview with the Regional Director of Health was facilitated by an interview guide to gather in-depth information relevant to the research question.^30^ The interview guide was also designed to cover the decision space elements as espoused in the survey instrument but was flexible to ensure further and more in-depth understanding of these key aspects were elicited. As regards the document analysis, a predesigned electronic form was specifically designed to serve as a guide to review policy documents and institutional records relevant to the 3 key domains of the decision space analytical framework. The tool aided review and analysis of policy outputs and the extent to which existing policy documents recognise and recommend actions to improve decision space at the sub-national levels.

The questionnaire and interview guide were pilot-tested in 2 districts prior to the actual data collection. The choice of these districts was purposefully sampled based on convenience. Consequently, the instruments were revised with minor changes made before utilised in the actual data collection process. Results from this pilot exercise did not form part of the results reported in this paper.

Regarding the questionnaire administration, the data collection process proceeded as follows. Field research assistants were recruited and trained about the study and the questionnaires administration procedure. Data collection followed a self-administration approach and so participants were handed out questionnaires for completion and returned to the research assistants. Verbal consent was sought from all participants before data collection. Participants were also made aware about the voluntary nature of the study. All sampled respondents completed the survey resulting in a 100% response rate. On the other hand, a face-to face interview was conducted in English by the first author using a semi-structured interview guide with the key informant, in this case the Regional Director of Health Service. The interview process was audio-recorded to ensure all essential information was captured without distortion. Prompts were frequently used to ensure clearer and further information was collected. Overall, the interview lasted for 50 minutes.

The quantitative data was analysed using a statistical software analysis package, SPSS version 17.0. Data were analysed descriptively and presented mainly in tables and graphs. Data from the interview was analysed through open coding by the first author. This process involved reading and rereading of interview transcript to code information relevant to the study question. Relevant codes from the interview were used to support or clarify some essential points or underscore some of the results from the cross-sectional survey.

Data for the document analysis were content analysed. For each of the human resource functional areas reported in the study, we analysed the existing relevant policy documents in the service. The document analysis targeted at policy documents relevant to human resource management. In all, 8 key policy documents were consulted as shown in Table S1 (see Supplementary file 1). We also consulted the annual reports from the regional level and national, program of works, including the legal provision (Act 525) that triggered the decentralisation reforms agenda in the health service.

## Results

### Basic Characteristic of Respondents

The study enlisted 164 participants comprising a regional director, 11 district directors, 6 medical directors/superintendents, 50 sub-district in-charges and 96 community health officers. Of these, whilst 1 participant (Regional Director) was interviewed using an interview guide, the remaining 163 participants made up of district directors, medical directors/superintendents, sub-district in-charges and community health officers were surveyed by means of a questionnaire. Of the 164 participants, 89 (54%) were females. Participants were most likely to be community health nurses (58%), younger than 25 years (66%), and would have held their current management position for less than 5 years (73%). Of the overall participants recruited for the survey, 28 (17%) were registered nurses or midwives, medical doctors comprised 5 (3%), physician/medical assistants were 9 (5%) and community health nurses were 96 (59%) respectively. The remaining 26% represented other categories including enrolled nurses. Table 2 shows the distribution of study respondents.

**Table 2:** Basic Characteristics of Respondents

**Characteristic**	**Total (N = 164)**	**%**
Age (y)		
<25	109	66
25-50	16	10
>50	39	24
Gender		
Female	89	54
Male	75	46
Category of managers		
Regional director	1	1
District/municipal director	11	7
Medical director/superintendent	6	4
Sub-district in-charge	50	30
Community health officers	96	58
Educational status		
Postgraduate	17	10
Bachelors/graduate	1	1
Diploma	28	17
Certificate	118	72
Basic professional category		
Medical doctor	5	3
Registered general nurse or midwife	28	17
Medical/physician assistant	9	5
Community health nurse	96	59
Enrolled nurse	13	8
Other	13	8
Years of experience in management position		
Less than 5 years	119	73
5 years or more	45	27

### Decision Space

#### Employment Management

The responses regarding the decision space which allows for the appointment, dismissal and determination of remuneration across 3 management levels (CHPS zones, sub-district, and district) within the regional health service is reported in Table 3. The responses pointed to a limited authority to manage these human resource functional areas. However, the regional director in the interview indicated that he had the prerogative to appoint a category of staff termed category D & E (staff grades for which secondary, post-secondary certificate or basic education is the entry requirements). To further explain the limitation to appoint or dismiss staff at the level of the regional health administration, the respondent had this to say; *“it is just a functional authority and not an initiating authority”* (key informant interview). This informant further explains that in the exercise of the discretion to appoint category D & E staff as stated above, it must be with financial clearance or approval from the ministry of finance and economic planning (MoFEP). A review of the appointments policy of the GHS corroborates the respondent’s views. Depending on the category of staff to be appointed or dismissed, the authority ranges from the head of state, through the GHS council, to the director responsible for human resource development of the GHS except for categories D & E indicated above for which the regional director exercises some level of autonomy.^32^ This policy does not however make provisions for appointing authority beyond the level of regional director such as the district, sub-district and CHPS zones level.

**Table 3:** Respondents View on Employment Management Functions

**Employment Management Functions**	**CHOs**			**Sub-district In-charges**			**District Directors/Medical Superintendents**			**Overall Total**		
	**Yes** **No. (%)**	**No** **No. (%)**	**Total** **No. (%)**	**Yes** **No. (%)**	**No** **No. (%)**	**Total** **No. (%)**	**Yes** **No. (%)**	**No** **No. (%)**	**Total** **No. (%)**	**Yes** **No. (%)**	**No** **No. (%)**
Appointment	0 (0.0)	96 (100.0)	96 (100.0)	0 (0.0)	50 (100.0)	50 (100.0)	0 (0.0)	17 (100.0)	17 (100.0)	0 (0.0)	163 (100.0)
Dismissals/sack	0 (0.0)	96 (100.0)	96 (100.0)	0 (0.0)	50 (100.0)	50 (100.0)	0 (0.0)	17 (100.0)	17 (100.0)	0 (0.0)	163 (100.0)
Salary determination	0 (0.0)	96 (100.0)	96 (100.0)	0 (0.0)	50 (100.0)	50 (100.0)	12 (70.6)	5 (29.4)	17 (100.0)	12 (7.4)	151 (92.6)

Abbreviation: CHOs, Community Health Officer.

Similar levels of autonomy was reported across the various management levels within the regional health service regarding salary determination as indicated in Table 3. While the CHPS zones and the sub-district levels report a lack of authority regarding this function, 12 respondents representing 70.6% of district and hospital managers indicate that they play a role such as recommending suspension of salary.

Evidence from the review of policy and programmatic documents also revealed that the regional administration plays a critical role in pay determination through pay processing for final approval by the MoFEP.^32^ However, regarding the pay levels, the existing pay policy which took effect from January 2010 vested the authority to determine pay levels in the Fair Wages and Salaries Commission of Ghana which is centrally managed at the national level.^33^ Because of this central determination of the pay levels exercised by this commission, none of the management levels within the region exercises discretion in that regard. Table 3 below provides details on this subject.

### Personnel Administration

The finding regarding decision space in personnel administration is mixed as indicated in Table 4. Across the 6 functions that constitute personnel administration, the CHPS zones report a lack of autonomy and discretion to carry out these functions. Though same can be reported for the sub-district managers regarding promotion, demotion, transfers/reassignment and sanctions, higher levels of autonomy and discretion have been reported at this level for personnel administration functions such as supervision (92%) and performance appraisal (88%). At the district and hospital levels, the autonomy and discretion for sanctions, supervision and performance appraisal of staff mainly resides with the district directors and medical superintendents as displayed in Table 4. Again, high levels of authority for promotions and transfers/reassignments have been reported for these categories of health managers in the region. On the contrary, only 11% of the respondents are of the view that they exercise autonomy and discretion over demotions. The regional health administration has equally reported mixed levels of discretion. In response to the level of authority and discretion exercised at the regional health administration regarding personnel administration the response included;

**Table 4:** Respondents Views on Personnel Administration Function

**Personnel Administrative Function**	**CHOs**			**Sub-district In-charges**			**District Directors/Medical Superintendents**			**Overall Total**		
	**Yes** **No. (%)**	**No** **No. (%)**	**Total** **No. (%)**	**Yes** **No. (%)**	**No** **No. (%)**	**Total** **No. (%)**	**Yes** **No. (%)**	**No** **No. (%)**	**Total** **No. (%)**	**Yes** **No. (%)**	**No** **No. (%)**
Promotions	0 (0.0)	96 (100.0)	96 (100.0)	0 (0.0)	50 (100.0)	50 (100.0)	14 (82.4)	3 (17.6)	17 (100.0)	14 (8.6)	149 (91.4)
Demotions	0 (0.0)	96 (100.0)	96 (100.0)	0 (0.0)	50 (100.0)	50 (100.0)	2 (11.8)	15 (88.2)	17 (100.0)	2 (1.2)	161 (98.8)
Transfers/Reassignments	0 (0.0)	96 (100.0)	96 (100.0)	0 (0.0)	50 (100.0)	50 (100.0)	15 (88.2)	2 (11.8)	17 (100.0)	15 (9.2)	148 (90.8)
Sanctions	0 (0.0)	96 (100.0)	96 (100.0)	0 (0.0)	50 (100.0)	50 (100.0)	17 (100.0)	0 (0.0)	17 (100.0)	17 (10.4)	146 (89.6)
Supervision	0 (0.0)	96 (100.0)	96 (100.0)	46 (92.0)	4 (8.0)	50 (100.0)	17 (100.0)	0 (0.0)	17 (100.0)	63 (38.7)	100 (61.3)
Performance appraisal	0 (0.0)	96 (100.0)	96 (100.0)	44 (88.0)	6 (12.0)	50 (100.0)	17 (100.0)	0 (0.0)	17 (100.0)	61 (37.4)	102 (62.6)

Abbreviation: CHOs, Community Health Officer.

*‘...it depends on the HR function and the level. For instance, while the authority to promote or demote category D* &* E staff in the region resides with the regional director, same cannot be said of other categories as that will have to be referred to the GHS HRDD [Human Resource Development Directorate] for consideration.…in terms of postings, supervision and performance appraisal of staff within the region, the regional director is the final authority. However, regarding sanctions, the regional director has limitations. For instance, action to dismiss a senior staff in the region can only be initiated at the regional level through recommendation to the director general [of GHS]. As such, the regional director does not have absolute discretion and authority on a matter like this*” [key informant interview].

A review of the GHS policy and guidelines on promotion provides some insight into the responses reported above. Per the said policy, no specific role or authority has been assigned to the CHOs and sub-district in-charges.^34^ On the other hand, the role of the district directors and medical superintendents has been specified to include assessing staff due for promotion and recommending such staff for the attention of the regional director. In respect of the regional director, he has been vested with authority to; promote category D&E in line with the prevailing promotion guidelines, assess category B & C staff below their last grades and submit the assessment reports of such staff to the HRDD of the GHS for further action.

In respect of postings which also includes transfers and reassignment within the regional health service, the policy and guidelines on postings stipulates among other things that, the regional director shall issue posting letters to the respective district directors who will in turn place such staff appropriately into needy health facilities within the district.^35^ Per the policy, the role of health facilities regarding these postings is to notify the posting authority of the assumption of duty or otherwise of the staff.^35,36^

Again, per the policy direction of the GHS, all staff are required to be supervised and appraised by their immediate superiors or managers.^37^ Responses from the survey as indicated in figure 3 largely confirms compliance of practice with policy except at the CHPS zones where respondents expressed a lack of authority or discretion for supervision and performance appraisal. Similarly, per existing policy, the GHS vests the authority for sanctions in the GHS council, the director general, directors at the various divisions, regions, districts as well as institutional heads^38^ Table 4 provides response on the role the respondents play regarding this subject.

### Staff Development

The decision space that have been reported from the survey in respect of in-service training, continuous professional development, post-basic training and fellowships across the various management levels in the study area are reported in Table 5. The survey indicates a lack of autonomy and discretion to manage these human resource functions at the CHPS zones and sub-district level. However, at the district level, 88% of the respondents reported that they exercise authority for post-basic training and continuous professional development while 52% indicated same for in-service training. Regarding fellowships, the general decision space reported point to a lack of or limited discretion and authority regarding this human resource function as shown in Table 5.

**Table 5:** Respondents Views on Staff Development Functions

**Staff Development Functions**	**CHOs**			**Sub-district In-charges**			**District Directors/Medical Superintendents**			**Overall Total**		
	**Yes** **No. (%)**	**No** **No. (%)**	**Total** **No. (%)**	**Yes** **No. (%)**	**No** **No. (%)**	**Total** **No. (%)**	**Yes** **No. (%)**	**No** **No. (%)**	**Total** **No. (%)**	**Yes** **No. (%)**	**No** **No. (%)**
In-service training	0 (0.0)	96 (100.0)	96 (100.0)	0 (0.0)	50 (100.0)	50 (100.0)	9 (52.9)	8 (47.1)	17 (100.0)	9 (5.5)	154 (94.5)
Continuous professional development	0 (0.0)	96 (100.0)	96 (100.0)	0 (0.0)	50 (100.0)	50 (100.0)	15 (88.2)	2 (11.8)	17 (100.0)	15 (9.2)	148 (90.8)
Post-basic training	0 (0.0)	96 (100.0)	96 (100.0)	0 (0.0)	50 (100.0)	50 (100.0)	15 (88.2)	2 (11.8)	17 (100.0)	15 (9.2)	148 (90.8)
Fellowships	0 (0.0)	96 (100.0)	96 (100.0)	0 (0.0)	50 (100.0)	50 (100.0)	16 (94.1)	1 (5.9)	17 (100.0)	16 (9.8)	147 (90.2)

Abbreviation: CHOs, Community Health Officer.

The key informant interview reported similar levels of decision space regarding human resource development functions.

*“The ultimate reference point in matters of fellowships and in-service training is the national level. But where the in-service training originates from the region, the regional director remains the officer in-charge…, for fellowship, my role is often to nominate or recommend eligible candidates for consideration at the national level. For post-basic training, recommendation is made to the HRDD for degree or higher qualification programs. However, with regard to other lower qualifications, the regional director has authority to grant these albeit this must be in line with the study leave policy in force at the time*…*in respect of CPDs [continuous professional development], they are profession-based and depends on the individual to be able to finance them or secure financing from their respective institutions to be able to attend.’* [key informant interview].

Per the GHS 2016 study leave policy and guidelines regarding the level of responsibility for managing the various personnel development programs,^39^ fellowships (both local and external) require national level approval although nomination or recommendation may be initiated from the regional level. Equally, the policy confers the approving authority for post- basic degree programs on the director of the HRDD of the GHS at the national level. The policy also corroborates the reports of the key informant regarding approving authority for non-degree awarding post-basic training and fellowships. Regarding CPDs, both the study leave policy and the service employee handbook^40^ are not explicit on the roles of the various management levels of the service on the subject matter.

Overall, the decision space allowed for the 12 human resource functions across the 4 management levels in the regional health service have been summarised into a matrix as shown in Table 6. This analysis was based on insights drawn from the questionnaires, the in-depth interview and document analysis. In the matrix, decision space has been categorised for each human resource function as narrow, moderate or wide. For a human resource function to be categorised narrow, it means that at that management level, there is no exercise of any discretion or authority regarding that function. Where the particular management level exercises some discretion albeit limited, the decision space is considered moderate. Finally, where absolute discretion or full authority is exercised over the function, the decision space at that management level is considered wide. To further illustrate this, in response to a question such as what role or authority you exercise over promotion of staff? Responses that indicate the exercise of no role or authority are rated narrow. Where the responses indicate that staff are either nominated, assessed and or recommended to a senior management level to be promoted, such responses are rated moderate. Finally, where the responses indicate that the promotion of staff is ultimately or absolutely carried out at a particular management level, the discretion at this management level is rated wide.

**Table 6:** Decision Space Across the Various Management Levels

**HR Functions**	**Range of Decision Space Across Various Management Levels**				
	**Regional Level**	**District Level**	**Sub-district level**	**Community Level**
	**Regional Health Administration**	**District Health Administration**	**Hospitals**	**Sub-district**	**CHPS Zone (Compound)**
Employment management					
Appointments	Narrow	Narrow	Narrow	Narrow	Narrow
Dismissals/firing	Narrow	Narrow	Narrow	Narrow	Narrow
Remuneration determination	Narrow	Narrow	Narrow	Narrow	Narrow
Personnel administration					
Promotions/demotions	Moderate	Moderate	Moderate	Narrow	Narrow
Transfers/reassignment	Wide	Moderate	Narrow	Narrow	Narrow
Sanctions	Wide	Moderate	Moderate	Narrow	Narrow
Supervision	Wide	Wide	Wide	Moderate	Narrow
Performance appraisals	Wide	Wide	Wide	Moderate	Narrow
Staff training and development					
In-service training	Moderate	Moderate	Moderate	Narrow	Narrow
Continuous professional development	Moderate	Moderate	Moderate	Narrow	Narrow
Post-basic training	Moderate	Moderate	Moderate	Narrow	Narrow
Fellowships	Moderate	Narrow	Narrow	Narrow	Narrow

Abbreviations: HR, human resource; CHPS, community-based health planning and services. Note: the following operational definitions were applied in contextualising the decision space analysis. Narrow: There exist no autonomy and discretion over this HR function at the respective management level. Moderate: Autonomy and discretion exercised for the HR function is partial/limited but not absolute, such as recommending action for the consideration of the next superior management level. Wide: Ultimate authority for the HR function resides at this level of management.

## Discussion

In this study, we sought to examine the level of decision space available in practice to subnational health authorities and the implications of such decision space on the management of human resource for health in Ghana. Whiles in the international literature decentralisation is expected to lead to the transfer of responsibility for the management of human resources to subnational units,^41,42^ the findings from this study suggest that most human resource functions are rather centralised. The study further revealed that discretion, limited as it may be, diminishes along the management continuum from the national to the community level. Decisions regarding hiring, firing or remuneration are highly centralised at the national level. This finding is in consonance with another study which conducted a comparative analysis of decision space between 4 countries – Ghana, Uganda, Zambia, and Philippines.^5^ Among the findings of the study, it was reported that Ghana has a unified hierarchical personnel structure in which decisions on contracting, hiring, firing and civil service benefit is centralised. The findings also resonate with a similar study of decentralisation and decision space in the Suva subdivision in Fiji which sought to examine how different functions under decentralised reforms have been decentralised.^15^ The study reported that decisions regarding established post, selection, salary determination, appointment, training, promotion and discipline are vested in a centralised public service commission. The consequence of the above could include staff shortage and adverse implications on productivity and the attainment of health sector objectives because it has been reported in other studies that, greater flexibility in hiring and firing at the local levels are associated with increased efficiency and quality of services.^16^ Furthermore, efficiency is likely to be adversely affected in the GHS because there is evidence to suggest that, the ability of local managers to hire, fire and provide specific incentives to employees improves efficiency.^43^

On the other hand, some benefits could be envisaged from the centrally controlled hiring, firing and salary determination responsibilities. These could include providing the opportunity to centrally manage the public sector wage bill, ensure equity in wage determination and equitable distribution of workforce.

It is however worth indicating that, the centralised nature of recruitment and appointment functions within the GHS could be attributable to the choice decentralisation adopted. That is, the choice of deconcentration over devolution or delegation as prescribed by ACT 525. As observed in the literature, deconcentration is unlikely to lead to the transfer of recruitment and appointment responsibility to the lower levels, rather devolution and delegation are more likely.^44^ As such, deconcentration may only lead to transfer of administrative responsibilities in line with national level directives for service delivery at the sub-national or lower levels of management.^45^ In accordance with this definition and as reported in this study, the level of decision space available in practice at the various management levels of the GHS is determined by national policy directives as evidenced by the various human resource policies reviewed in this study. These directives prescribe the autonomy and discretionary levels regarding each human resource function at every management level which has informed current practice. Again, the justification for the central control of employment in the health sector is not far-fetched. According to the MoH,^46^ 57.3% of total health spending and 90% of the recurrent budget was used for personnel compensation for the year 2013. Therefore, an attempt to maintain control of the health sector budget requires maintaining control over the human resource budgets. A study by Bach^47^ equally cited the desire to maintain tight control of public sector wage bill as the rational for the reluctance of government to delegate significant autonomy for pay determination to lower organisation levels.

Further, in relation to personnel administration, the study reported mixed levels of decision space and this could present significant and varying implications on human resource management within Ghana’s health sector. The evidence that the regional level exhibits wide discretion and autonomy in all aspects of personnel administration whilst, the district level only exercises wide discretion regarding performance appraisal and supervision suggests an emphasis on output and personnel control without equal attention on other personnel functions such as motivation and staff development. On the other hand, given that part of the personnel administration function is to manage the performance of staff through the provision of technical supervision and performance appraisal, the wide range of discretion exercised at this level as per this study could be considered appropriate and likely to improve the performance of the health sector. Several studies in other jurisdictions support this position because, it has been observed that, by simplifying the personnel administration function through the reduction in bureaucracy, and less overload and congestion in the channels of communication and administration, improvement in the quality and quantity of public services is often realised.^42,44,48-50^ It is worth noting however that, some studies have reported that decentralisation in many situations confuses supervisory responsibilities, diminishes technical supervision capacity and reduces supervisory visits.^51,52^ This study does not entirely agree with this position as findings from the present study present a contrasting picture. The hierarchical structure of the GHS as depicted in Figure equally defines accountability and responsibility relationships which to a large extent prevents the confusing roles in supervision and performance appraisal as reported in other studies.

While the findings generally point to a wide discretion in the performance assessment such staff appraisal, same cannot be said of the reward system. Ultimate authority for promotions and demotion are mainly centralised. The regional and district health services, as well as the hospitals’ roles in promotion, are largely advisory and subject to the approval of the central authority. The seeming gap in the discretion and authority to assess performance versus providing rewards (promotion/demotion) can adversely affect workforce performance. Based on this, it is reasoned that, once health personnel do not see a direct link between their performance and the consequent reward, they may not be motivated to improve productivity in the hope of a reward. Suffice it to state that, the discretion exercised at the regional and district levels regarding promotion is significant because it forms the basis upon which decisions at the central national level regarding these HR functions are made. It must also be indicated that, the level of decision space in practice for personnel administration largely reflects national policy directives except for CHPS zones where there is an indication of limited autonomy and discretion for staff supervision and appraisal contrary to national level policy directives. Additionally, it is also evident that, the decision space for personnel administration is a derivative of deconcentration which limits administrative authority to national level directives.

In the health sector, personnel development is an important function because it provides an opportunity for health workers to provide quality healthcare to meet the communities’ changing healthcare needs through updating their professional knowledge, skills, values and practices.^53^ The level of discretion exercised at the local level on staff capacity development is, therefore, an opportunity to tailor personnel development to meet local preferences and the changing health needs of the communities. Therefore, the limited discretion regarding this functional area reported in this study could affect the performance of the health sector and how responsive training and staff development programs could be to the respective local communities. As revealed by this study, regional, as well as district health service, are limited to nominating or recommending candidates for such opportunities. An important aspect of this moderate decision space at both the regional and district level is that it ensures that the inputs of the lower levels are considered in granting approval for all forms of personnel development programs. It goes without saying however that, opportunities which are developed centrally may not be with recourse to local level needs and preferences. The bureaucratic procedures involved could equally have an impact on the timeliness of such personnel development programs and how useful they may be to the health sector.

We situate our discussion within some limitations of the study. Decentralisation encompasses 3 dimensions viz decision space, individual and institutional capacities and local accountabilities. This study however focussed on only decision space notwithstanding the fact that studies have underscored the utility of exploring the 3 dimensions together as a way of understanding the interaction among these dimensions.^54,55^ The implications of decentralisation on local capacities and accountability as a whole has therefore not been addressed by this study. The study is also descriptive and as such does not provide a mechanism to measure the direct impact of the findings on human resource management. Additionally, capturing and categorising the exact range of choice available at the local management level is an inherent limitation of the decision space approach. The study is also cross-sectional, and the results point to the period of research in 1 region which could vary over time and across regions. Further, this study was conducted in 1 of 10 regional health services and thus results should be interpreted and applied with caution as this does not represent the overall situation in Ghana. Nonetheless, we contend that the results from this study offer useful insights towards healthcare sector reforms to ensure greater decision space for managers to operate. We acknowledge this present study presents some strengths despite the limitations. Foremost, it is the first of its kind to examine the effect of decentralisation reform on a specific functional area such as human resource management in the Ghanaian context. Secondly, the study disaggregates the human resource function into specific functional areas and thereby provides a well-focussed analysis of the implications of decentralisation on these specific human resource functional areas and the implications thereof on the entire health sector. Through a comprehensive document review, the study also provides relevant context to responses obtained from the survey and thereby informs an in-depth analysis and discussion on the subject. As such, the findings and conclusions provide further insights on the effect of decentralisation reform in the health sector for both policy purposes and the research community.

## Conclusion

This study concludes that though decentralisation presupposes the transfer of responsibility and authority for the management of human functions from the central national level to the respective sub-national management levels, findings from the study point to the contrary as the decentralisation of the human resource function is more apparent than real. Management authority regarding core human resource functions are centralised at the GHS national level and as such, provides very limited discretion and autonomy at the subnational levels. This situation is understood to be a direct outcome of the form of decentralisation (deconcentration) adopted in the health sector in Ghana. Deconcentration as a form of administrative decentralisation relies on national policy directives to determine the decision space available at subnational levels which is largely the practice in Ghana’s health sector. We also conclude that, management discretion for the management of human resources diminishes along the management continuum from the national to the community level. Further, the study also revealed an apparent disjoint between the wide decision space for performance assessment through technical supervision and performance appraisal and a rather limited discretion for providing incentives or rewards. Decentralisation was however found to have led to greater autonomy and discretion in performance assessment and personnel administration in the GHS. It was also found to provide the potential for improved salary administration and equity in human resource distribution. To realise the benefits of decentralisation in respect of human resource management for health, there is the need to give further meaning to decentralisation in the GHS by increasing the decision space in practice at the subnational level. This will require a revision of ACT 525 which provides the legal basis for the form of decentralisation practiced in the health sector. Human resource policy revision is also required in the GHS to empower local levels with the relevant decision-making autonomy and discretion as well as capacity to manage human resource for health. We conclude by recommending that further studies consider a framework for measuring the direct effects of decentralisation and should include other functional areas beyond the human resource management function. Most importantly, we recommend that further studies should assess all 3 dimensions of decentralisation namely decision space, individual and institutional capacities and local accountability together to provide an overall insight into the implications of decentralisation on human resource as well as other management functional areas.

## Acknowledgements

We acknowledge Dr. Insah Baba, School of Business, Wa Polytechnic, Wa, Ghana for his significant inputs into the study design.

## Ethical issues

Written approval was sought from Upper West Regional Health Administration and from the various district and medical directors/superintendents of health before the study was conducted.

## Competing interests

The authors declare they have no competing interest.

## Authors’ contributions

The study was designed by both authors. AMS analysed, interpreted and wrote the first draft. LB contributed to the interpretation and writing of the manuscript. Both authors reviewed, read, and approved the final manuscript.

## Authors’ affiliations

^1^Ghana Health Service, Upper West Regional Health Directorate, Wa, Ghana. ^2^Regional Institute for Population Studies, University of Ghana, Legon-Accra, Ghana. ^3^School of Allied and Public Health, Faculty of Health Sciences, Australian Catholic University, Sydney, NSW, Australia.

## Supplementary files

Supplementary file 1 contains Table S1.

## Key messages

**Implications for policy makers**

- The effect of decentralisation on the management of human resource for health is largely dependent on the form of decentralisation adopted and the context within which it is applied.
- To realise the benefits of decentralisation, there is the need to transfer authority and discretion for managing human resource from centralised bodies to localised levels.
- Limited or lack of decision space at the localised level regarding personnel recruitment, remuneration, retention, distribution and development could result in adverse implications including shortage of staff.
- Wide decision space for performance assessment at the localised level should be accompanied with increased authority and discretion to provide rewards.

**Implications for public**

Decentralising the management of human resource in the health sector has the potential to improve quality, efficiency, equity, innovation, and access to healthcare delivery as well as enhance local participation in the health sector decision-making. To realise these benefits, the authority and discretion for managing human resource should be transferred to the localised levels or units. Where there is limited or no transfer of this authority and discretion to the localised units as reported in this study, adverse human resource implications could result. These may include the persistent shortage of staff, high staff turnover, inadequate technical skills, inequitable distribution of staff and inconsistency between performance and rewards system.

## Supplementary files

Supplementary file 1 contains Table S1.
