# Supplementary material for: Decentralisation and Management of Human Resource for Health in the Health System of Ghana: A Decision Space Analysis
Source: Int J Health Policy Manag. 2018 Sep 23;8(1):28–39. doi: 10.15171/ijhpm.2018.88 (PMC6358646; doi:10.15171/ijhpm.2018.88)
Supplement: Supplementary file 1 — contains Table S1. [file ijhpm-8-28-s001.pdf]

Supplementary File 1

Table S1. Resources Consulted for the Document Analysis

SN	Category	Key Documents Reviewed	Others
	**Employment Management**<br>1. Appointments<br>2. Dismissals/firing<br>3. Remuneration determination	1. Ghana Health Service – Human Resource Development Directorate 2007, policy and guidelines on appointments, GHS, Accra Ghana<br>2. Fair wages and salaries commission<br>3. Ghana Health Service/Human Resource Development Directorate 2006, instructional guide for conducting induction and orientation for newly appointed/assigned staff in the Ghana health service, Accra Ghana<br>4. Ghana Health Service – Human Resource Development Directorate 2006, Employee hand book, GHS, Accra Ghana<br>5. Ghana Health Service 2003 Code of conduct and disciplinary procedures GHS, Accra, Ghana.	1. Ghana Health Service 2011, 2010 annual report, GHS, Accra, Ghana<br>2. Ghana Health Service 2012, 2011 annual report, GHS, Accra, Ghana<br>3. Ghana Health Service 2013, 2012 annual report, GHS, Accra, Ghana<br>4. Ghana Health Service 2015, 2014 annual report, GHS, Accra, Ghana<br>5. Ghana Health Service 2011, 2010 annual report, GHS, Accra, Ghana<br>6. Ghana Health Service 2017, 2016 annual report, GHS, Accra, Ghana<br>7. Upper west regional health service 2011, 2010 annual report, RHA, Wa, Ghana<br>8. Upper west regional health service 2012, 2011 annual report, RHA, Wa, Ghana<br>9. Upper west regional health service 2013, 2012 annual report, RHA, Wa, Ghana
	**Personnel Administration**<br>1. Promotions/Demotions<br>2. Transfers/Reassignments<br>3. Sanctions<br>4. Supervision	1. Ghana Health Service – Human Resource Development Directorate 2007, policy and guidelines on promotions, GHS, Accra Ghana	

	5. Performance Appraisals	2. Ghana Health Service – Human Resource Development Directorate 2007, policy and guidelines on postings, GHS, Accra Ghana<br>3. GHS 2016: Guidelines for Supportive Supervision in the Health Sector, GHS, Accra<br>4. Ghana Health Service 2003 Code of conduct and disciplinary procedures GHS, Accra, Ghana.<br>5. Ghana Health Service – Human Resource Development Directorate 2006, Employee hand book, GHS, Accra Ghana	10. Upper west regional health service 2014, 2013 annual report, RHA, Wa, Ghana<br>11. Upper west regional health service 2015, 2014 annual report, RHA, Wa, Ghana<br>12. Upper west regional health service 2016, 2015 annual health report, RHA, Wa, Ghana<br>13. Ministry of Health, n.d. the Ghana health sector 2011 program of work: going beyond strategy to action – accelerating activities towards the millennium development goals, MOH, Accra, Ghana<br>14. Ministry of Health n.d. 2015 program of work: working together towards universal coverage- accelerating the momentum for attaining health related MDGS, MOH, Accra Ghana.<br>15. Ministry of Health 2008, The Ghana health sector 5years program of work 2007-2011: creating wealth through health, MOH, Accra, Ghana<br>16. Ghana Health Service n.d. GHS monitoring and evaluation plan 2014-2017, GHS, Accra Ghana<br>17. Republic of Ghana, 1996, Ghana Health Service and Teaching Hospitals Act, 1996 (ACT 525, 1996)
	**Staff Training and Development**<br>1. In-service training<br>2. Continuous professional development<br>3. Post basic training<br>4. Fellowship	1. Ghana Health Service – Human Resource Development Directorate 2007, policy and guidelines on counselling, GHS, Accra Ghana<br>2. GHS 2016, Guidelines for award of study leave in 2016, GHS Accra, Ghana	
